# Environmentally controlled magnetic nano-tweezer for living cells and extracellular matrices

**DOI:** 10.1038/s41598-020-70428-w

**Published:** 2020-08-10

**Authors:** Christian Aermes, Alexander Hayn, Tony Fischer, Claudia Tanja Mierke

**Affiliations:** Faculty of Physics and Earth Science, Peter Debye Institute of Soft Matter Physics, Biological Physics Division, University of Leipzig, Linnéstr. 5, 04103 Leipzig, Germany

**Keywords:** Nanoscale biophysics, Biological physics

## Abstract

The magnetic tweezer technique has become a versatile tool for unfolding or folding of individual molecules, mainly DNA. In addition to single molecule analysis, the magnetic tweezer can be used to analyze the mechanical properties of cells and extracellular matrices. We have established a magnetic tweezer that is capable of measuring the linear and non-linear viscoelastic behavior of a wide range of soft matter in precisely controlled environmental conditions, such as temperature, CO_2_ and humidity. The magnetic tweezer presented in this study is suitable to detect specific differences in the mechanical properties of different cell lines, such as human breast cancer cells and mouse embryonic fibroblasts, as well as collagen matrices of distinct concentrations in the presence and absence of fibronectin crosslinks. The precise calibration and control mechanism employed in the presented magnetic tweezer setup provides the ability to apply physiological force up to 5 nN on 4.5 µm superparamagnetic beads coated with fibronectin and coupled to the cells or collagen matrices. These measurements reveal specific local linear and non-linear viscoelastic behavior of the investigated samples. The viscoelastic response of cells and collagen matrices to the force application is best described by a weak power law behavior. Our results demonstrate that the stress stiffening response and the fluidization of cells is cell type specific and varies largely between differently invasive and aggressive cancer cells. Finally, we showed that the viscoelastic behavior of collagen matrices with and without fibronectin crosslinks measured by the magnetic tweezer can be related to the microstructure of these matrices.

## Introduction

Force spectroscopy has evolved as a powerful tool to study of the mechanical properties of single molecules^1–4^, assemblies of macromolecules^5–7^ and even entire living cells^8–11^. The invention of optical tweezer has been the initial pioneering event, in which dielectric microscopic objects are probed by focused light laser beam^12,13^.

Optical tweezers may cause a heating of the sample that seems to impact the mechanical properties of living cells. At the same time, optical stretchers are cost intensive and can only probe purely spherical objects, such as deadhered and suspended cells. Due to these shortcomings, the first magnetic tweezers were invented in 1996 and first used to determine the elasticity of supercoiled DNA^14^. More precisely, a pair of moveable permanent magnets is positioned above a glass flow chamber containing DNA molecules that has been mounted on an inverted microscope and can be employed to probe paramagnetic beads in a magnetic field gradient. In order to measure the elasticity of soft biomaterials, such as cells and reconstituted collagen matrices, we employed and further developed a magnetic tweezer technique.

In this study we present a magnetic tweezer setup that is suitable for probing a wide variety of different viscoelastic materials. For this, we investigated two different human breast carcinoma cell lines (MDA-MB-231 and MCF-7 cells), two different mouse embryonic fibroblast cell lines (PINCH-1 knock-out (PINCH-1^−/−^) cells and controls (PINCH-1^fl/fl^), as well as 3D collagen gels with and without fibronectin crosslinking. The aim of this study was to demonstrate that the presented setup is suitable to probe differences in the non-linear viscoelastic properties between different cell lines as well as different models for extracellular matrices. Using this setup, it is possible to establish a linkage between structural parameters of extracellular matrix networks and their respective mechanical properties. In addition, the presented setup is equipped with a surrounding incubation chamber to precisely control the environmental conditions during measurements, such as temperature, humidity and carbon dioxide. Earlier studies investigating cellular mechanics employed heated microscopic stages to control the temperature^15–17^ or do not control the temperature at all^18–20^. Due to the strong influence of external parameters on cellular mechanics^21,22^, the employment of a surrounding incubation chamber with precisely controlled environmental conditions is therefore a major improvement compared to earlier implementations of the magnetic tweezer setup.

Although there are many setups dealing with several permanent magnetic poles^23,24^, the highest forces are generally obtained with a one-pole microneedle geometry that can be either achieved using permanent magnets^25^ or electromagnets with soft iron cores^16,26–30^. Our setup for the magnetic tweezer consists of a single solenoid that contains a high-permeability soft iron core, which is connected to a conventional remote-controlled micromanipulator. The force in this non-linear force distance relationship was controlled by tracking the bead-core distance during the measurement and calibrated by the viscous drag approach^29,31^. If necessary, the solenoid current can be adapted automatically.

The setup can exert forces in the range of a few nanonewtons, which is sufficient to deform living cells. Living cells are revealed to be relatively soft with Youngs moduli of hundreds Pascal (Pa) up to one kilo-Pascal. Cell mechanics are important for many physiological functions, including cell proliferation, cell adhesion, cell migration, and cell contraction.

Measurements of cell mechanical properties have been performed in a high-force or large-deformation regime that revealed contradictory findings. Cells have been reported to stiffen^30,32–43^, or on the contrary fluidize and yield^30,44,45^ or to behave completely linearly and constantly after application of external stretching forces over a certain timescale^46^. However, these different results can rely on different experimental conditions and cell models utilized. Hence, we performed for this study cell and matrix mechanical measurements together employing magnetic tweezers in an environmental chamber. The environmental chamber ensures stable conditions during the measurements, such as controlled CO_2_ supply and temperature. Other studies that employ magnetic tweezers for live cell measurements rely on a heated microscope stage for temperature control^29,39,47,48^ which may induce temperature gradients and thus affect the mechanical properties of the cells. In fact, we analyzed time- and force-dependent viscoelastic properties of adherent cells under more controlled conditions at large forces, ranging from 1 to 5 nN. Moreover, we determined the mechanical properties of 3D extracellular matrices, in which these cells possess different capacities to invade^49–52^. Since ILK has been shown to affect the deformability and invasion of cells into 3D collagen fiber matrices^53,54^, we analyzed the effect of PINCH-1, on cell mechanics.

We observed an increase of the stiffness and a decrease or increase of the power law exponent with increasing force application repeatedly in cancer cells, such as MDA-MB-231 and MCF-7 cells, as well as in mouse embryonic fibroblasts such as PINCH-1 knock-out (PINCH-1^−/−^) cells and controls (PINCH-1^fl/fl^). PINCH-1 (synonymously termed Lims 1) represents a LIM-only domain protein and can form a ternary complex with integrin-linked kinase (ILK) and parvin (termed IPP complex). The complex is downstream of the integrins. More precisely, PINCH-1 can bind to actin through a LIM-domain and actin-binding proteins such as epithelial protein lost in neoplasm (EPLIN)^55^.

In addition, 3D extracellular matrix mechanics, such as matrix stiffness (elastic modulus), can be determined with the magnetic tweezer using collagen-bound superparamagnetic beads coated with fibronectin. We found that denser 3.0 mg/ml collagen matrices were significantly stiffer than looser 1.5 mg/ml collagen matrices. Both matrices represent still confinements for cancer cell and fibroblast migration. Finally, our findings seem to be important for a quantitative phenotypic characterization of biological processes, such as cell migration and invasion.

## Material and methods

### Cells and cell culture

MDA-MB-231 human breast cancer cells (malignant and highly invasive) were cultured in low glucose (1 g/l) Dulbecco’s modified Eagle’s medium (DMEM, Biochrom, Berlin, Germany) supplemented with 10% fetal bovine serum (FBS) (Biochrom, Berlin, Germany) and 1% penicillin/streptomycin (P/S, Biochrom, Berlin, Germany). MCF-7 human breast cancer cells (non-malignant and lowly invasive), PINCH-1 cells carrying a Lox-P-flanked PINCH-1 gene (PINCH-1^fl/fl^ cells), and PINCH-1 knock-out cells (PINCH-1^−/−^) mouse embryonic fibroblasts were cultured in high glucose (4.5 g/l) DMEM supplemented with 10% FBS and 1% P/S^56^. Cells were passaged at a confluence of about 80%. These two cell-types were kindly provided by Dr. Reinhard Fässler (MPI Munich).

For magnetic tweezer measurements, 4.5 µm super paramagnetic beads with an epoxylated surface (Dynabeads M450, Sigma Aldrich) were coated with human fibronectin (50 µg/ml, Sigma Aldrich). In detail, beads were first washed in PBS. Then, the fibronectin was added. The beads were then centrifuged for about 24 h at 37 °C and about 8 g according to the coating protocol provided by the manufacturer. Thereafter, the beads were washed twice in PBS and stored at 8 °C. Prior to measurements, beads were rigorously agitated by a vortex mixer in order to break up any clusters that may have formed during storage.

About 24 h before a measurement, cells were seeded onto 35 mm culture dishes (about 10^5^ cells per dish). Cells were then incubated at 37 °C and 5% CO_2_. On the day of the measurement, about 5·10^4^ fibronectin coated beads were added to the cells. The cells were then incubated at 37 °C and 5% CO_2_ for 20 min. Thereafter, cells were washed twice with PBS to remove any unbound beads. Cells were measured no longer than 40 min with the magnetic tweezer. After that time, beads were usually internalized by the cells. About 30 cells can be measured during the 40-min interval^57^.

Only cells with a single bound bead were investigated. The bead-needle distance was chosen in such a way that the maximal possible force was greater than the desired force. When placing the needle, great care was taken not to poke the cell with the needle tip.

### 3D collagen matrix analysis

For the investigation of extracellular matrix mechanics, we used 1:2 mixtures of collagen type I rat tail and bovine skin. Two collagen gels of different concentrations (1.5 mg/ml and 3.0 mg/ml) were prepared as described previously^49,53,58,59^. In detail, collagen gels were prepared on ice to prevent polymerization. Collagen R (4 mg/ml rat collagen type I, Serva, Heidelberg, Germany) and collagen G (4 mg/ml bovine collagen type I, Biochrom, Berlin, Germany) were mixed in a 1:2 ratio and added to a phosphate buffered solution. The phosphate buffered solution (pH 7.4, ionic strength of 0.7, 200 mM phosphate) consisted of a mixture of sodium dihydrogen phosphate, disodium hydrogen phosphate in ultrapure water. For the preparation of gels crosslinked with fibronectin, 25 µg/ml of fibronectin (Sigma Aldrich) were added. To maintain the correct collagen concentration, the amount of water was reduced by the volume of added fibronectin. 100 µl collagen were pipetted into a 35 mm culture dish. The gels were polymerized at 37 °C, 95% humidity and 5% CO_2_ for 2 h. Then, the gels were rinsed with 3 ml PBS and stored at 37 °C, 95% humidity and 5% CO_2_ until measurement. For magnetic tweezer measurements, about 5·10^4^ fibronectin coated beads were added to the gels on the day of the measurement. The gels were then incubated for about 20–30 min at 37 °C and 5% CO_2_. During the measurements in the magnetic tweezer, collagen gels were kept at 37 °C and 5% CO_2_ enriched air.

### Matrix topological properties

Collagen matrices were prepared as described above. In the next step, 500 µl of the cooled, unpolymerized collagen monomer solution were added into each well of a pre-cooled ibidi 24-well µ-Plate. Collagen hydrogels were polymerized by putting the well-plates in an incubator at 37 °C 95% humidity for 2 h. These polymerized collagen gels were rinsed three times using PBS at room temperature. Subsequently, the collagen matrices were fluorescently stained using 20 µg/ml 5(6)-Carboxytetramethylrhodamine *N*-succinimidylester (TAMRA-SE) (Sigma Aldrich, Cat. No: 21955) for 24 h. Finally, the stained matrices were washed three times using PBS and stored in 1 ml PBS at 4 °C. Imaging of the stained samples was performed using a confocal laser scanning microscope (Leica TCS SP8, Mannheim, Germany).

For pore-size determination, a 40 × NA/1.10 water immersion objective was used to record 3D image cubes with an edge length of 100 µm. Images are recorded with a resolution of 2048 pixels in x- and y-direction. For each cube, about 400 stacks are recorded. A deconvolution was applied to these images using the Huygens Essentials v16.10 software (Scientific Volume Imaging B.V., Hilversum, Netherlands). For the analysis, pixels are rescaled to 37.5% in the x- and y-direction^60^. The final images used therefore have a resolution of 0.13 px/µm in the x–y-plane and 0.25 µm/px in the z-direction.

The pore-sizes of these 3D images were determined using a custom-built python program^60^. This algorithm includes a precise segmentation of collagen fibrils and residual pore detection applied to the fluid phase of this segmentation, as seen in Fig. 7e.

### Magnetic tweezer technique

#### Measurement procedure

The heating unit was turned on at least 1 h before the measurement to ensure a stable temperature in the incubation chamber surrounding the magnetic tweezer. The CO_2_ supply was switched on at least 20 min before the measurement. The culture medium was flushed directly with 5% CO_2_ enriched air by placing the tube of the CO_2_ supply directly on the rim of the cell culture dish.

Three different force protocols are available. The simplest force protocol is the application of a single force pulse with a constant magnitude. Another available force protocol is a sequence of consecutive pulses with equal magnitudes and duration. Consecutive pulses are separated by a relaxation period of equal duration, during which the force is returned to zero. The third force protocol is a staircase-like sequence of force pulses. In this protocol, the force is increased after each step by a constant amount until a maximum set force is reached. Each step in this protocol has the same duration.

#### Data analysis

The creep response J(t) was defined as the ratio of strain ε and stress σ. The stress in the magnetic tweezer measurements was estimated as the ratio of the applied force and the contact area between bead and the cell. As an approximation of the contact area, the bead cross section area A = πr^2^ was used. Therefore, σ = ΔF/(πr^2^). The strain was defined as the displacement of the bead divided by the radius of the bead, i.e. ε = d(t)/r.

Thus, the displacement curves were scaled by πr/ΔF to get the creep compliance.

For the application of a sequence of pulses, the creep compliance at t ≥ t_n_ is a superposition of ongoing creep responses from t < t_n_,

1 $$ J\left( {t \ge t_{n} } \right) = \mathop \sum \limits_{i = 0}^{n} J\left( {t - t_{i} } \right) + J\left( {t - t_{n} } \right) $$

Rheological parameters were derived by fitting the creep compliance with the Kelvin–Voigt model and a weak power law:

2 $$ J\left( t \right) = J_{0} \left( {1 - e^{{ - {\raise0.7ex\hbox{$t$} \!\mathord{\left/ {\vphantom {t \tau }}\right.\kern-\nulldelimiterspace} \!\lower0.7ex\hbox{$\tau $}}}} } \right) $$

3 $$ J\left( t \right) = J_{0} \left( {{\raise0.7ex\hbox{$t$} \!\mathord{\left/ {\vphantom {t {t_{0} }}}\right.\kern-\nulldelimiterspace} \!\lower0.7ex\hbox{${t_{0} }$}}} \right)^{\beta } $$

For both models, the inverse of J_0_ represents a measure for the stiffness of the cell. The relaxation time τ in the Kelvin–Voigt model was the viscosity divided by the elastic modulus. In power law rheology, the exponent β was the cell fluidity, which represents the viscoelastic state of the cell. For example, a β = 0 indicates a purely elastic behavior while a β = 1 displays a purely viscous behavior. The parameter t_0_ was a reference time and set to one. Therefore, J_0_ was the creep compliance evaluated at t_0_ = 1 s.

Statistical analysis revealed that cell stiffness J_0_^−1^ followed a log-normal distribution. Therefore, the average cell stiffness was calculated as the geometric mean. However, the cell fluidity followed a normal distribution. Thus, the average cell fluidity was calculated as the arithmetic mean.

### Statistical assessment

For significance tests, a Welch’s t-test was performed. Additionally, a Bonferroni correction was applied to account for errors introduced by multiple testing. Due to the log-normal distribution of the stiffness values, the significance test was carried out on the log-transformed values. Results were considered significant for *p* < 0.05 and are marked by asterisks (*p < 0.05, **p < 0.01 and ***p < 0.001) in the respective figures.

## Results

### Establishment and further development of the magnetic tweezer setup equipped with a 3D environmental chamber

**Figure 1:**
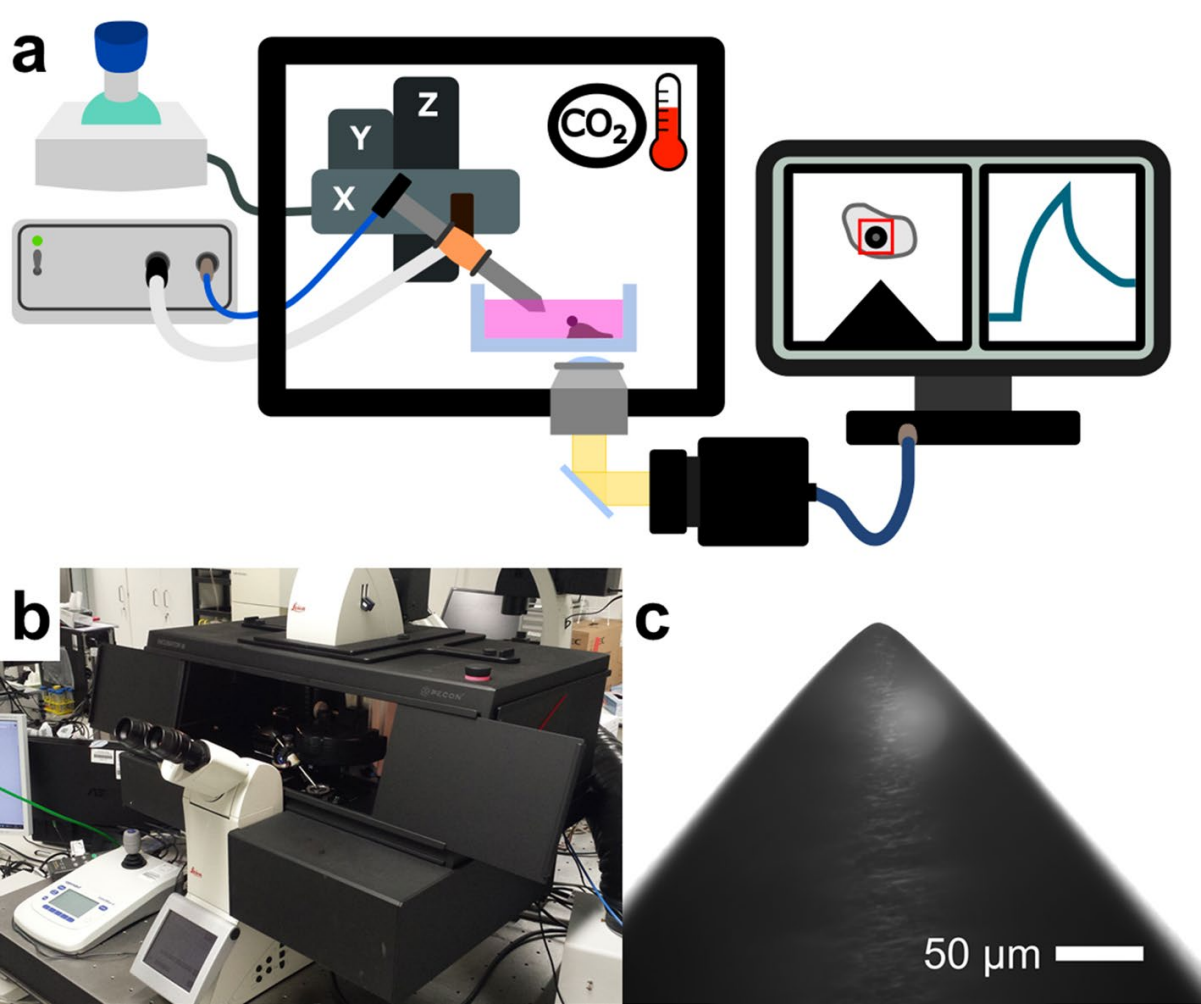
(**a**) Schematic image of the magnetic tweezer setup created with Inkscape^87^. The magnetic field was generated by a coil with a ferromagnetic core made from µ-metal. A custom-built voltage driven current supply was connected to the coil to generate the magnetic field. The magnetic tweezer was connected to a micromanipulator that allowed precise placement of the needle tip close to the specimen. The whole setup was mounted on an inverted microscope and enclosed by an incubation chamber with temperature and CO_2_ control. Cells with super paramagnetic beads bound to them were placed in a petri dish with cell medium. The petri dish was then placed under the microscope. A CMOS camera captured live images of the bead's deflection under an acting magnetic force from the tweezer. This deflection was then tracked live over time and analyzed by a custom written program. (**b**) Picture of the magnetic tweezer setup. (**c**) To generate a high gradient field, the core was shaped conically at its tip, forming a needle. The tip had an opening angle of about 35° and a radius of curvature of about 10 µm. Scale bar is 50 µm.

Since the cell mechanical properties play an important role in cell migration and invasion of cancer cells and fibroblasts, we established and further developed a magnetic tweezer setup, in which the cells can be measured under controlled CO_2_, humidity and temperature. A schematic overview of the magnetic tweezer setup is presented in Fig. 1a. The magnetic tweezer setup was built on top of an inverted microscope (DMI8, Leica). Brightfield images were recorded with a CMOS camera (Orca Flash 4.0 V3, Hamamatsu) at a data acquisition rate up to 40 Hz. The setup was equipped with a heatable incubation chamber. Additionally, the chamber can be filled with 5% CO_2_ enriched air to allow an extended measurement of living cells. The whole setup was placed on top of an anti-vibration desk for more stable tracking conditions during measurements (Fig. 1b).

The magnetic tweezer consisted of a coil with a soft ferromagnetic core made from µ-metal. The coil was provided with current from a custom-built power supply. The tip of the core was shaped in a conical geometry with an opening angle of about 35° and a radius of curvature of about 10 µm (Fig. 1c). The shape of the tip was generated through mechanical grinding with very fine sandpaper (3,000 and 5,000 grid).

At the blunt end of the tip, a Hall-probe was attached to monitor the magnetic field strength during measurements. For a precise positioning of the tip in front of a cell, the magnetic tweezer was attached to a 3D micromanipulator (Injectman, Eppendorf). The magnetic tweezer and microscope were both controlled by a custom-written LabVIEW (National Instruments) program.

**Figure 2:**
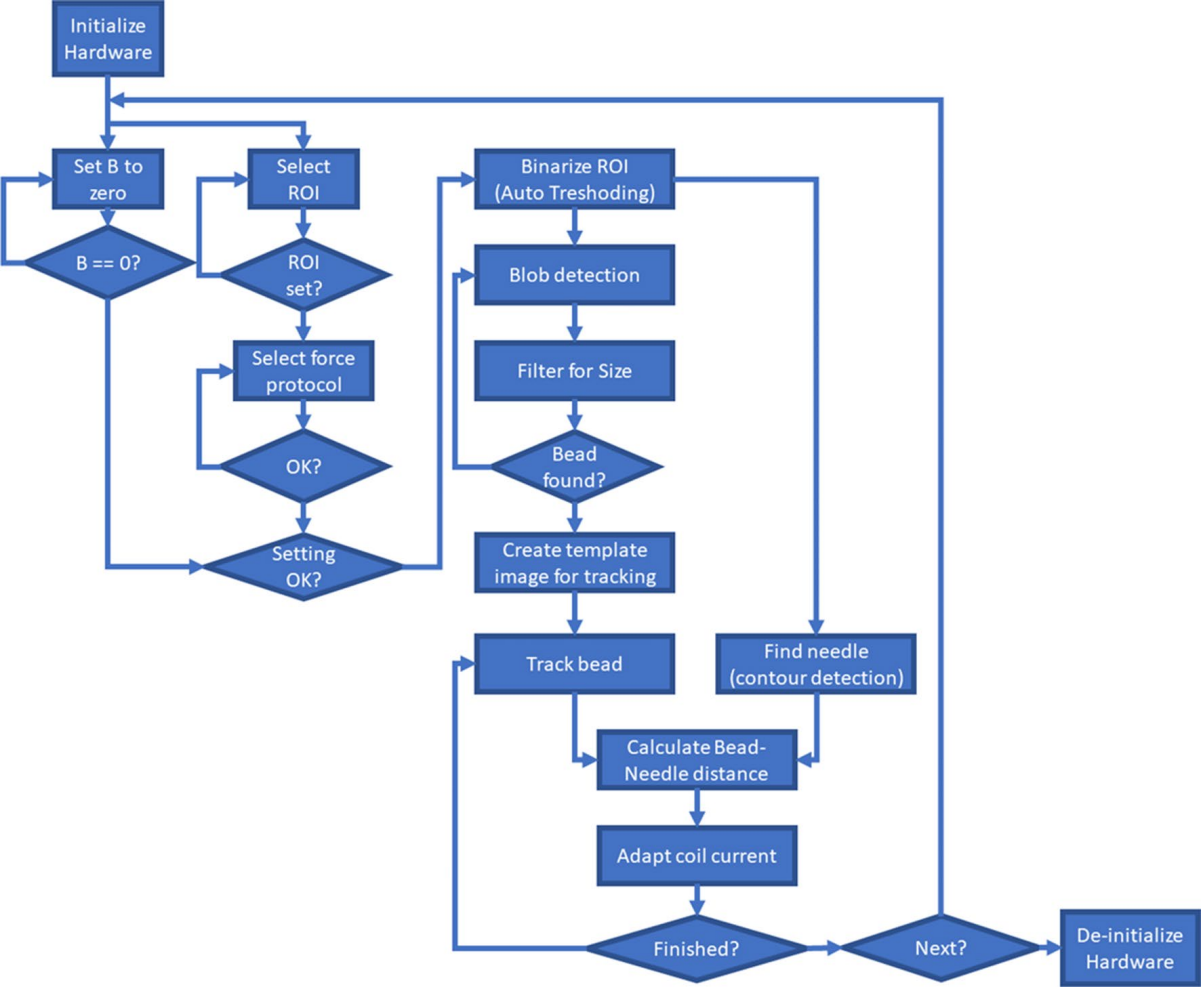
Flow chart of the measurement software contains all important steps: After initialization of the hardware, the remnant magnetization of the core was automatically set to zero. The image shows a ROI selected for bead and needle position tracking. During the measurement, the coil current was continuously adapted based on the bead-to-needle distance to ensure a constant force. When a measurement was completed, the program was either set up for the next measurement or the hardware was de-initialized and finished.

The program is presented in Fig. 2 in the form of a flow chart. The program runs as follows: First, the hardware (camera, microscope stage, and current supply) was initialized. When the coil current was turned off, the core retained a miniscule magnetization that was measured by the Hall-probe. This remnant magnetization was automatically cancelled by applying a small coercive current to the solenoid. The magnitude of the coercive current was calculated based on a running average of the magnetic field strength. Image analysis was performed on a region of interest (ROI) of the image. After the ROI and a measurement protocol were selected, the ROI was binarized via automatic thresholding. A blob detection was performed on the binarized image to detect particles. To identify beads the particles were then filtered by size. Once a bead was found, a template image of that bead was generated for an image cross-correlation tracking. During tracking, the needle contour was extracted from the binary image. Based on the bead position and the needle contour, the bead-needle distance was calculated as shortest Euclidean distance. Based on this distance, the coil current was continuously adapted to apply a constant force to the bead.

### Force calibration of the magnetic tweezer

The setup was calibrated by a viscous drag approach. Super paramagnetic beads (M450, Dynabeads) with a diameter of 4.5 µm were diluted in two different poly-dimethyl siloxane solutions with known viscosities of 3,000 cSt and 5,000 cSt, respectively. The calibration liquid had a final bead concentration of about 3·10^5^ ml^−1^. The needle tip was placed at a distance of about 120 µm from a randomly selected bead. Beads were chosen so that they were at least 200 µm from any walls in order to avoid wall effects. Then, a sequence of pulses with constant current was applied to the solenoid. Each pulse and relaxation phase between two consecutive pulses lasted 2 s. In current-on phases, the bead was pulled toward the needle tip. In current-off phases, the bead drifted slightly due to settling of the fluid. This drifting motion was subtracted from the movement of the bead in the current-on phase. The force acting on the bead was then calculated via Stoke’s equation from the drift-corrected velocity. The velocity of the bead was determined via numerical differentiation of its displacement.

Calibration measurements were carried out for currents between 0.01 and 3 A. Therefore, the force acting on a bead can be determined as a function of bead-needle distance and coil current. Calibration curves were fitted with a power law with a current dependent power law exponent^29^.

4 $$ F\left( {I,d} \right) = F_{0} d^{c\left( I \right)} $$

5 $$ c\left( I \right) = \frac{{c_{1} }}{{1 + c_{2} \cdot {\exp}\left( {c_{3} I} \right)}} $$

**Figure 3:**
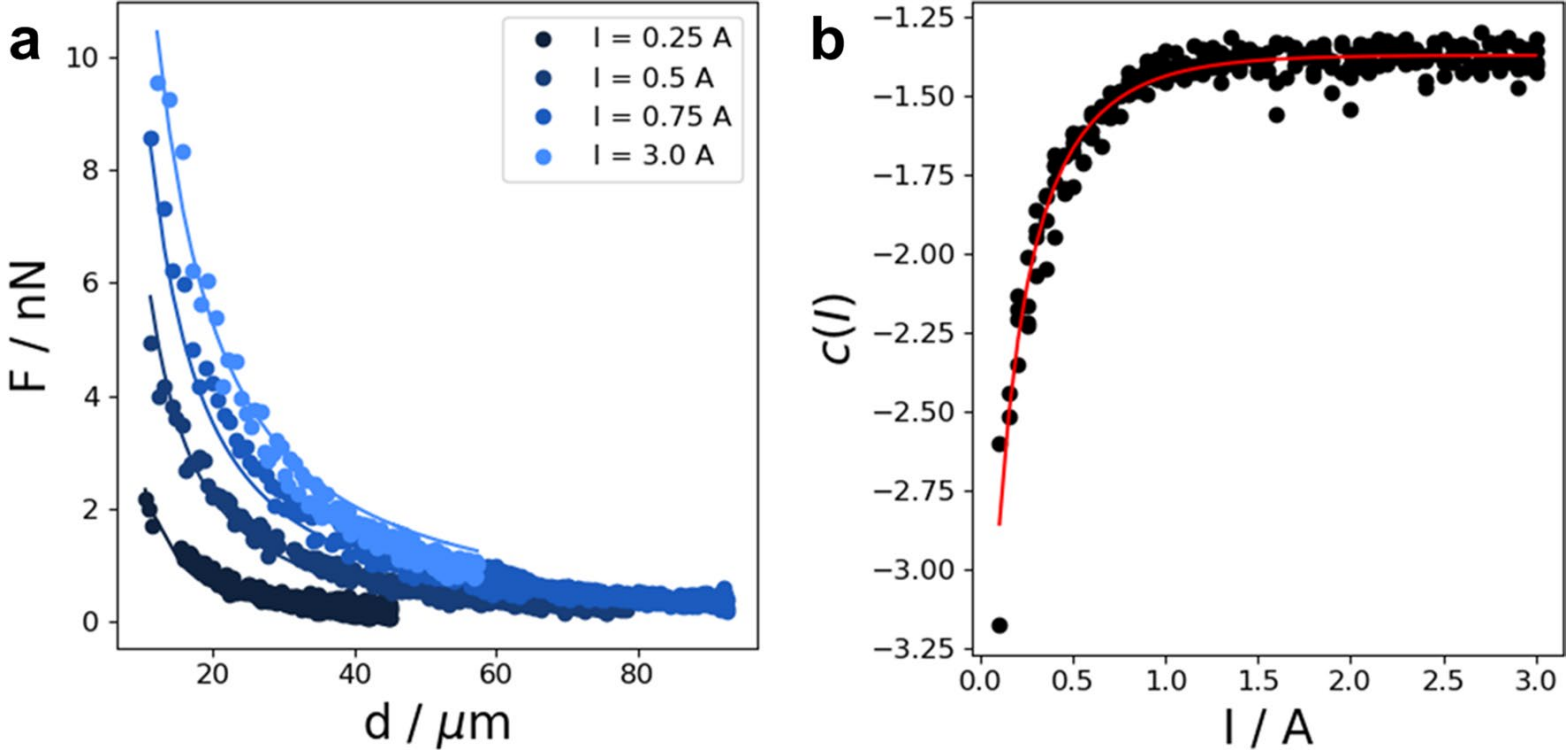
Force calibration for precise control during measurements. (**a**) Exemplary distance dependence of the force on 4.5 µm beads is presented for four different currents. Force–distance curves were fitted with a power law. (**b**) The power law exponent of the fit was current-dependent and was fitted with Eq. 5.

Exemplary calibration curves and the fit to Eq. 4 are presented in Fig. 3a. The force dependent power law exponent from the fit of Eq. 4 to the calibration curves is shown in Fig. 3b and fitted with Eq. 5. Due to the highly non-linear force–distance relation, a force-feedback loop was implemented to keep the force on a bead constant during measurements. As the bead moved closer to the needle tip, the solenoid current was reduced according to the calibration:

6 $$ I\left( {F,d} \right) = \frac{1}{{c_{3} }}{\ln}\left( {\frac{1}{{c_{2} }}\left( {c_{1} \cdot \frac{{{\ln}\left( d \right)}}{{{\ln}\left( {F/F_{0} } \right)}}} \right) - 1} \right) $$

The force generated by the magnetic tweezer was directly proportional to the current applied to the solenoid up to the magnetic saturation of the core material. The saturation magnetization was reached at a coil current of about 1 A. At this current, the magnetic tweezer can apply a force of about 11 nanonewtons to a bead at a distance of 10 µm.

### Cell mechanics of cancer cells with different invasive potential

**Figure 4:**
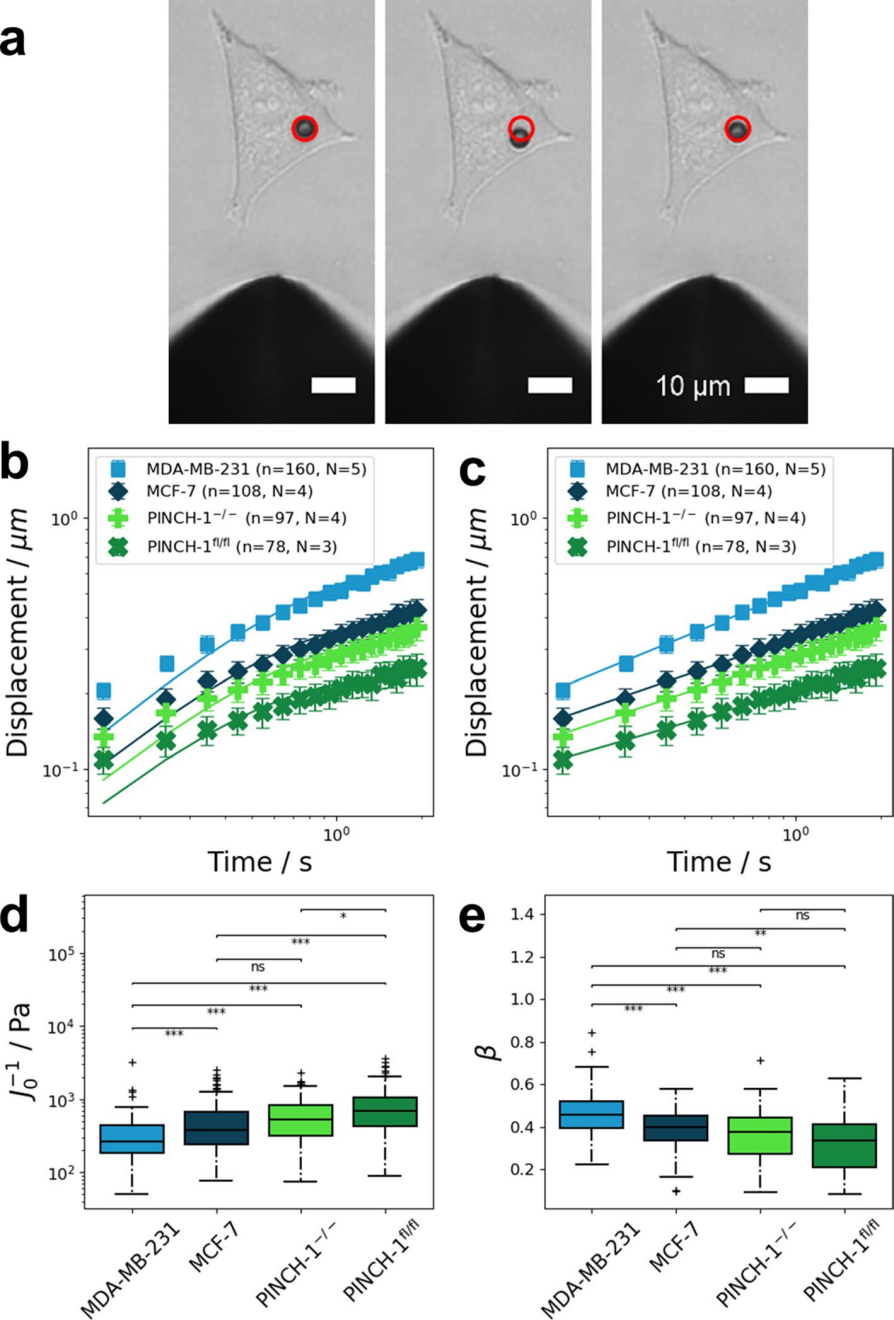
Results of measurements on cells with a single force pulse with 1 nN amplitude and 2 s duration. (**a**) A representative brightfield image shows a 4.5 microm super paramagnetic bead coupled to an MCF-7 cancer cell. The red circle in all images marked the initial position of the bead. Scalebars are 10 microm. Left: Bead position just before the force was turned on. Middle: Bead position after 2 s of force application. Right: Bead position after 2 s of relaxation after the force is turned off. (**b**) The averaged creep response of MDA-MB-231 (n = 160, N = 5), MCF-7 (n = 108, N = 4), PINCH-1^−/−^ (n = 97, N = 4), and PINCH-1^fl/fl^ (n = 78, N = 3) cells over time was fitted with the Kelvin–Voigt model. Especially at lower time scales, the Kelvin–Voigt model fails to predict the creep curves correctly. This may be seen from the R^2^ values of the fit (MDA-MB-231: R^2^ = 0.9634; MCF-7: R^2^ = 0.9298; PINCH-1^−/−^: R^2^ = 0.9175; PINCH-1^fl/fl^: R^2^ = 0.8892). (**c**) The same averaged creep response was fitted with a weak power law. The creep response for all cell lines closely followed a weak power law over time. The R^2^ values of the power law fit are consistently higher than for the Kelvin–Voigt model for all cell lines (MDA-MB-231: R^2^ = 0.9978; MCF-7: R^2^ = 0.9978; PINCH-1^−/−^: R^2^ = 0.9968; PINCH-1^fl/fl^; R^2^ = 0.9968). The different cell lines displayed distinct differences in their creep response. (**d**) Stiffness values of MDA-MB-231, MCF-7, PINCH-1^−/−^, and PINCH-1^fl/fl^ cells obtained from the weak power law fit. E) Cell fluidity (power law exponent β) of MDA-MB-321, MCF-7, PINCH-1^−/−^, and PINCH-1^fl/fl^ cells. *p < 0.05, **p < 0.01, ***p < 0.001 and *ns* not significant.

The mechanical properties of MDA-MB-231, MCF-7, PINCH-1^−/−^ cells and PINCH-1 control cells carrying a LoxP-flanked PINCH-1 gene (PINCH-1^fl/fl^ cells) were measured by applying a constant force of one nanonewton over 2 s. Thus, the needle tip was placed at a distance between 20 and 50 µm away from a bead bound to a cell. All measurements were carried out at 37 °C and 5% CO_2_. A representative MCF-7 cell with a coupled bead is presented in Fig. 4a. The position of the bead coupled to the cell was tracked over time within the recorded images. From the position of the bead, the displacement curve was calculated. The curves were fitted with the Kelvin–Voigt model (Fig. 4b) and a weak power law (Fig. 4c). However, as evident from Fig. 4c, the displacement curves closely followed a weak power law. The R^2^ values of the power law fit are above 0.99 for all four cell lines (MDA-MB-231: R^2^ = 0.9978; MCF-7: R^2^ = 0.9978; PINCH-1^−/−^: R^2^ = 0.9968; PINCH-1^fl/fl^: R^2^ = 0.9968). In contrast, the Kelvin–Voigt model fitted the data less well, as evident from the lower R^2^ values (MDA-MB-231: R^2^ = 0.9634; MCF-7: R^2^ = 0.9298; PINCH-1^−/−^: R^2^ = 0.9175; PINCH-1^fl/fl^: R^2^ = 0.8892). Therefore, the average stiffness of cells was determined from the prefactor J_0_ of the power law. The measured stiffness values are presented in Fig. 4d. Human breast cancer cells, such as the highly invasive MDA-MB-231 and weakly invasive MCF-7 cells^49,61,63^, displayed pronounced differences in their stiffness J_0_^−1^. For MDA-MB-231 cells, an average cell stiffness of 277 ± 28 Pa (n = 160, N = 5) was measured. MCF-7 cells were on average stiffer and displayed an average stiffness of 420 ± 68 Pa (n = 108, N = 4). Both PINCH-1 cell lines, such as PINCH-1 knock out (PINCH-1^−/−^) cells and PINCH-1^fl/fl^ control cells were on average stiffer than both human breast cancer cell lines. The weakly invasive^55,64^ PINCH-1^−/−^ cells displayed an average stiffness of 503 ± 71 Pa (n = 97, N = 4). For highly invasive^55,64^ PINCH-1^fl/fl^ cells, an average stiffness of 688 ± 124 Pa (n = 78, N = 3) was measured.

The cell fluidity β is presented in Fig. 4e. For MDA-MB-231 cells, the average cell fluidity was 0.46 ± 0.02 (n = 160, N = 5). MCF-7 cells were less fluid with an average cell fluidity of 0.39 ± 0.02 (n = 108, N = 4). PINCH-1^−/−^ displayed a fluidity of 0.36 ± 0.02 (n = 97, N = 4). PINCH-1^fl/fl^ cells were the most elastic cell type with an average fluidity of 0.32 ± 0.03 (n = 78, N = 3).

### Effect of repeated constant force and increased force applications on cell mechanics, such as stiffness and fluidity

**Figure 5:**
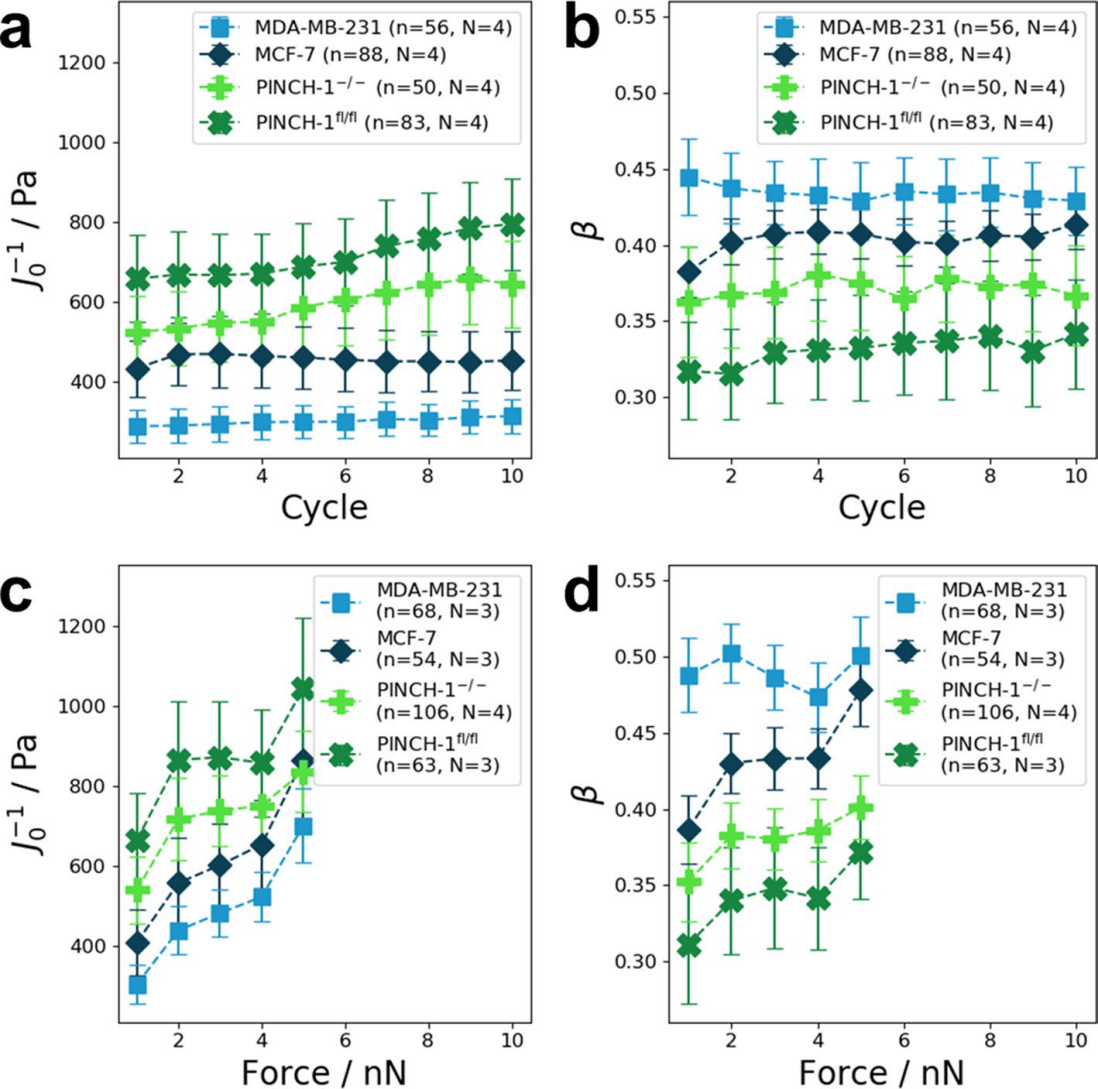
(**a**) The application of ten consecutive pulses lead to a stiffening in PINCH-1^−/−^ (n = 50, N = 4) and PINCH-1^fl/fl^ cells (n = 83, N = 4), while the stiffness of MDA-MB-231 (n = 56, N = 4) and MCF-7 cells (n = 88, N = 4) was not affected by the cycle number. Each force pulse had a magnitude of 1 nN and lasted for 2 s. Between two consecutive pulses, the force was returned to 0 for 2 s. (**b**) Cell fluidity (power law exponent β) was unaffected by the cycle number for all four cell lines. (**c**) Stiffness values obtained from a weak power law fit to the creep curves in response to the application of a staircase-like force sequence. For the staircase-like sequence, the initial force was 1 nN. Each step in the sequence lasted for 2 s. After each step, the applied force was increased by 1 nN. The last step in the sequence had a magnitude of 5 nN. MDA-MB-231 (n = 68, N = 3), MCF-7 (n = 54, N = 3), PINCH-1^−/−^ (n = 97, N = 4), and PINCH-1^fl/fl^ (n = 78, N = 3) cells displayed a significant increase in stiffness with the applied force. (**d**) The power law exponent β of MDA-MB-231, MCF-7, PINCH-1^−/−^, and PINCH-1^fl/fl^ cells also displayed a force dependence.

After the application of a single force pulse, cells recovered only partially. Therefore, the mechanical properties of cells after the first force application seem to be altered. To investigate this, a sequence of ten consecutive pulses was applied to the cells. Each pulse lasted 2 s and was followed by a 2 s relaxation period during which no force was applied. The resulting average cell stiffness J_0_^−1^ during creep is presented in Fig. 5a as a function of cycle number. For MDA-MB-231 cells (n = 56, N = 4 and MCF-7 cells (n = 88, N = 4), the average cell stiffness was relatively independent of cycle number. In contrast, PINCH-1^fl/fl^ control cells (n = 83, N = 4) and PINCH-1^−/−^ cells (n = 50, N = 4) displayed a pronounced strain-hardening, as indicated by the increase of J_0_^−1^ with increasing cycle number. However, the cell fluidity, the power law exponent β, did not depend on cycle number for all cell lines (Fig. 5b).

The force dependence of cellular mechanics was investigated by applying a staircase-like sequence of force pulses. The force increased from 1 to 5 nN in increments of 1 nN. Each force step lasted 2 s. After 2 s, the force was increased by 1 nN. For all cell lines, an increase in stiffness was observed with increasing force (Fig. 5c). The amount of stress stiffening appeared to be cell line dependent. The stiffness of both cancerous cell lines displayed a two-fold increase in stiffness between 1 and 5 nN (Fig. 5c). In contrast, the stiffness of both PINCH-1^fl/fl^ control cells and PINCH-1^−/−^ cells was only increased about 1.5-fold. For MDA-MB-231 cells, the stiffness increased by a factor of 2.31 ± 0.48 (n = 68, N = 3) (Fig. 5c). Stress stiffening was less pronounced in MCF-7 cells. The average cell stiffness measured for MCF-7 cells increased by a factor of 2.12 ± 0.61 (n = 54, N = 3). PINCH-1^−/−^ cells displayed an increase in cellular stiffness by a factor of 1.55 ± 0.31 (n = 106, N = 4). PINCH-1^fl/fl^ cells displayed a similar behavior with an average increase in stiffness by a factor of 1.57 ± 0.39 (n = 63, N = 3) (Fig. 5c). Cell fluidity also displayed a force dependence (Fig. 5d). For PINCH-1^fl/fl^ cells, the average power law exponent β increased by a factor of 1.20 ± 0.18 (n = 63, N = 3). PINCH-1^−/−^ cells displayed an average increase in fluidity by a factor of 1.14 ± 0.10 (n = 106, N = 4). The average power law exponent β for MCF-7 cells was increased by a factor of 1.24 ± 0.09 (n = 54, N = 3), whereas for MDA-MB-231 cells (n = 68, N = 3), no significant stress-induced fluidization was observed in the investigated force range (Fig. 5d).

### Matrix mechanics of loosely and densely confined 3D extracellular matrices

**Figure 6:**
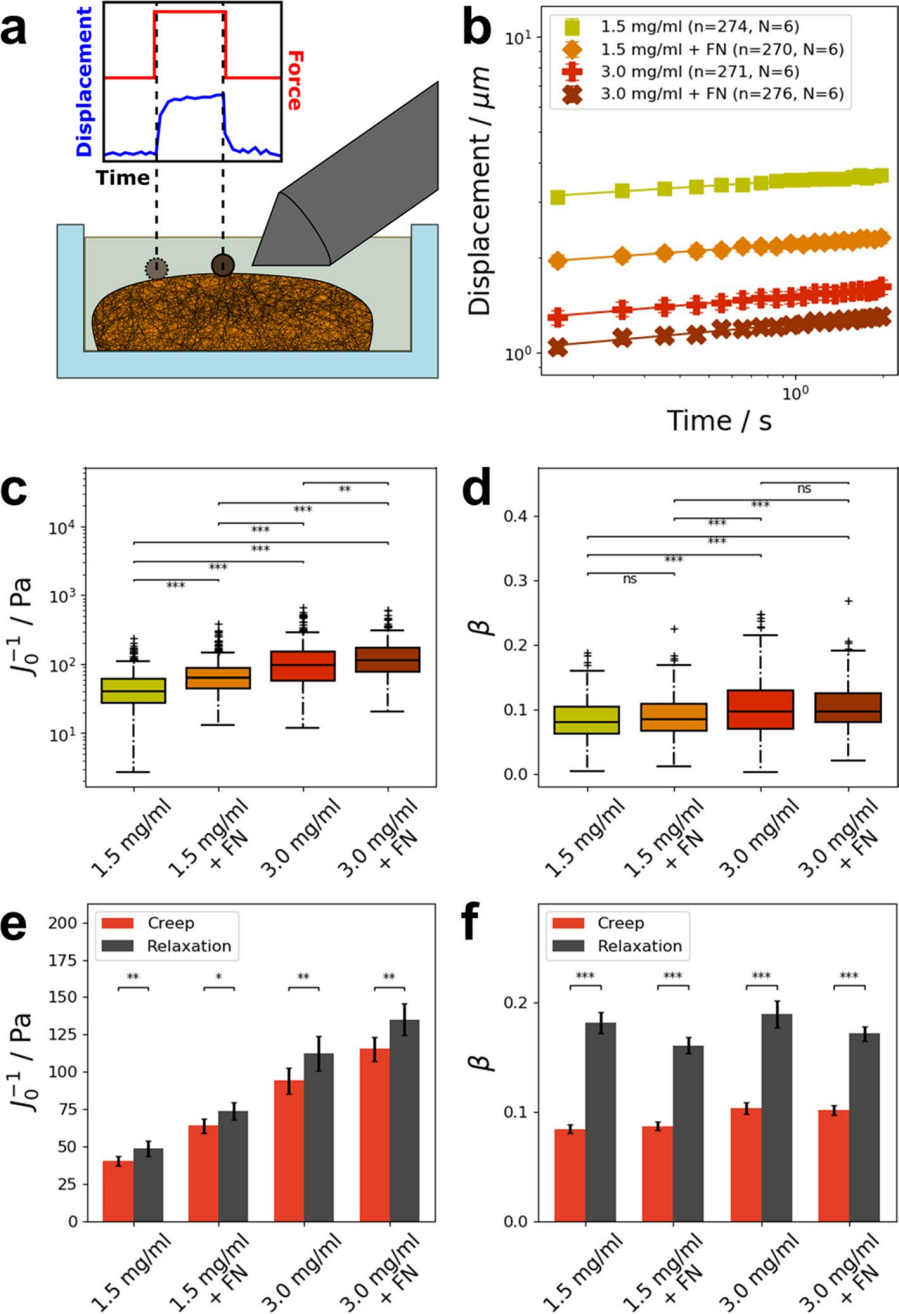
Magnetic tweezer measurements of ordinary collagen gels and gels crosslinked with fibronectin (FN). (**a**) Schematic representation of the measurements created with Inkscape^87^. The gels were polymerized in a cell culture dish and submerged in PBS. Super paramagnetic beads (with a diameter of 4.5 µm) were coupled to the surface of the collagen matrix. A constant force of one nanonewton was applied for 2 s to displace the beads. (**b**) Averaged displacement curves of beads coupled to collagen gels with a 1.5 mg/ml (n = 275, N = 6) and 3.0 mg/ml (n = 274, N = 6) type I collagen concentration (a 2 to 1 mixture of bovine to rat collagen) and both collagen gels crosslinked with fibronectin (1.5 mg/ml + FN: n = 270, N = 6; 3.0 mg/ml + FN: n = 276, N = 6). The displacement closely followed a power law. Note: Error bars in the curves are smaller than the respective symbols. (**c**) Stiffness values of the collagen gels. Fibronectin increased the stiffness of collagen gels significantly. (**d**) Power law exponent β from the fit. Crosslinking with fibronectin did not affect the power law exponent β. (**e**) Comparison of the stiffness values during creep and relaxation for the different collagen gels. The significantly higher stiffness during relaxation indicates incomplete recovery behavior. **f** The relaxation of collagen gels displayed a more fluid-like behavior compared to the preceding creep. *p < 0.05, **p < 0.01, ***p < 0.001 and *ns* not significant.

The magnetic tweezer can also be used to measure the mechanical properties of collagen gels. For this, we used mixtures of 1:2 rat and bovine collagens with different concentrations (1.5 mg/ml and 3.0 mg/ml). Additionally, the effect of crosslinking with fibronectin was investigated. The gels were probed with a single force pulse of one nanonewton magnitude and 2 s duration. For this, the needle tip was placed 40 to 60 µm away from a bead coupled to the surface of the collagen gel. A schematic representation of the measurement geometry is presented in Fig. 6a. Similar to cells, the creep response of the matrices was fitted with a weak power law (Fig. 6b).

The resulting stiffness values are presented in Fig. 6c. For 1.5 mg/ml gels, an average stiffness of 40 ± 3 Pa (N = 6, n = 275) was measured. Collagen gels with a concentration of 3.0 mg/ml were significantly (*p* < 0.001) stiffer with an average stiffness of 93 ± 9 Pa (N = 6, n = 274). Crosslinking of the gels with fibronectin significantly increased the stiffness of both 1.5 mg/ml and 3.0 mg/ml gels. For 1.5 mg/ml gels crosslinked with fibronectin, an average stiffness of 64 ± 5 Pa (N = 6, n = 270) was measured. The stiffness of 3.0 mg/ml gels crosslinked with fibronectin increased to 115 ± 8 Pa (N = 6, n = 276).

The power law exponent β of the fit is presented in Fig. 6d. Collagen gels with a concentration of 1.5 mg/ml displayed an average power law exponent β of 0.084 ± 0.004 (N = 6, n = 275). For 1.5 mg/ml gels crosslinked with fibronectin, a similar average power law exponent of 0.087 ± 0.004 (N = 6, n = 270) was observed. The power law exponent β of 3.0 mg/ml gels was significantly higher (*p* < 0.001) with an average value of 0.103 ± 0.005 (N = 6, n = 274). Crosslinking with fibronectin did not affect the average power law exponent β of 3.0 mg/ml collagen matrices, as these gels displayed a similar value of 0.102 ± 0.04 (N = 6, n = 276). These results indicate that the power law exponent β is not dependent on the crosslinking with fibronectin, whereas the stiffness corresponds to it, since the crosslinked matrices were significantly stiffer, when the concentration of collagen was increased. In summary, when these collagen matrices were crosslinked via the fibronectin, both matrices increased pronouncedly in stiffness, whereas the fluidities were not significantly changed.

After switching the force off, the bead displacement was recorded for another 2 s in order to reveal the relaxation behavior of the matrices. The displacement of the bead during relaxation was fitted with a superposition of the ongoing and a new power law creep process according to Eq. 1. The resulting values for the stiffness J_0_^−1^ and matrix fluidity (power-law exponent β) were compared to the values of the initial creep. The recovery of all collagen gels was generally incomplete, as indicated by the significantly higher stiffness values reported in Fig. 6e. These results indicate a strain-stiffening effect during force application for all four probes. The creep recovery also showed a more fluid-like behavior compared to the preceding creep for all examined collagen gels (Fig. 6f). When comparing the relaxation behavior between the pure collagen and the fibronectin crosslinked collagen gels, we detected a significantly higher stiffness in fibronectin crosslinked collagen gels in the relaxation phase and increased fluidity for both collagen concentrations compared to their individual creep phase stiffness. These results indicate a strain stiffening effect after fibronectin facilitated collagen fiber alignment.

### Topological properties of loosely and densely confined 3D extracellular matrices

**Figure 7:**
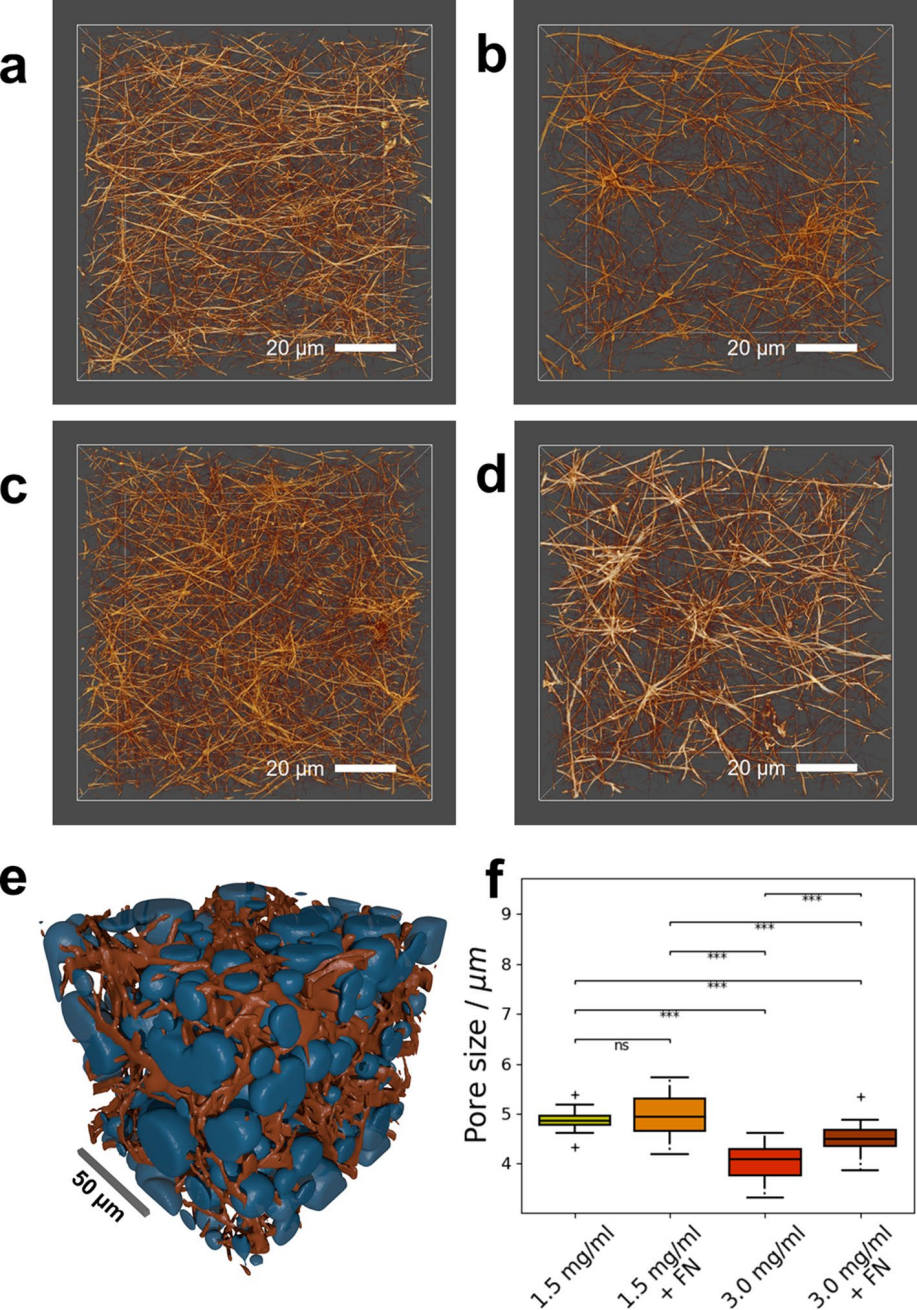
Determination of pore sizes for the investigated collagen gels. (**a**) Representative 3D image of a 1.5 mg/ml collagen gel. (**b**) 3D image of a representative fibronectin-crosslinked 1.5 mg/ml collagen gel. (**c**) 3D image of a representative 3.0 mg/ml collagen gel. (**d**) 3D image of a 3.0 mg/ml collagen gel crosslinked with fibronectin. Scale bars in (**a**)–(**d**) are 20 µm. (**e**) Visualization of the pore-fitting algorithm. Spheres (blue) are fitted into the fluid phase of the binary segmentation of the collagen gel (orange) to determine the pore size. The scalebar is 50 µm. Segmentation and pore data were determined as described previously^60^. Pore representations were created by drawing the respective spheres into a binary matrix using custom written Python software. Segmentation and pore representations were exported to STL surface models using Fiji^88^, and subsequently rendered using Blender^89^. (**f**) Pore sizes of the investigated collagen gels. 1.5 mg/ml collagen gels (N = 6) have a bigger pore size than 3.0 mg/ml gels (N = 6) due to the lower concentration of collagen fibers in the gel. Crosslinking with fibronectin increases the pore size of 3.0 mg/ml collagen gels (N = 6) but has no significant effect on the pore size of 1.5 mg/ml collagen gels (N = 5). ***p < 0.001 and *ns* not significant.

In order to connect the measured mechanical properties of the investigated collagen matrices to their respective topological properties, the gels were imaged using a confocal laser scanning microscope (CLSM). Representative 3D images of the matrices are presented in Fig. 7a–d. A precise segmentation algorithm was applied to the data and spheres were fitted into the 3D pores to determine the pore size of the respective collagen gels. A visualization of this fitting procedure is presented in Fig. 7e. The results of this pore size analysis are presented in Fig. 7f. Pores of 1.5 mg/ml collagen gels are significantly larger than those of 3.0 mg/ml gels, irrespective of treatment with fibronectin. For 1.5 mg/ml collagen matrices, fibronectin did not affect the detected pore size. However, 3.0 mg/ml collagen gels crosslinked with fibronectin display significantly bigger pores than 3.0 mg/ml gels without fibronectin. This result is qualitatively in line with the observation from the representative images in Fig. 7a–d. There, the fibers of pure 1.5 mg/ml gels (Fig. 7a) appear to be distributed more evenly within the volume, whereas fibers of fibronectin-crosslinked 1.5 mg/ml gels tend to form more nodes (Fig. 7b). The same behavior can be observed in 3.0 mg/ml collagen matrices, where the fibers of the non-crosslinked matrices (Fig. 7c) are distributed more evenly than the fibers of the crosslinked matrices (Fig. 7d). Generally, the images suggest that the fibers of fibronectin-crosslinked matrices seem to be more aligned than in non-crosslinked gels. This alignment is more pronounced in 3.0 mg/ml matrices compared to 1.5 mg/ml matrices due to the higher concentration of collagen fibers that can potentially be crosslinked.

## Discussion

The presented magnetic tweezer setup was suitable to probe a variety of different soft viscoelastic materials. With the current setup, displacements of about 40 nm were detectable. Therefore, materials with stiffness values up to 3.5 kPa can be measured when applying a constant force of 1 nN.

In order to accurately apply the force to the cells, a designated force-feedback loop has been incorporated into the design of the magnetic tweezer. This feedback loop accounts for the highly non-linear force–distance by finely tuning the magnetic field strength and subsequently the force.

To further ensure a precise measurement of mechanical properties of cells, the needle tip has been carefully shaped so that it can be placed in the same focal plane as a bead coupled to a cell using the precise 3D-micromanipulator. This prevented out of focus displacement of the beads when a force was applied. The forces generated by the magnetic tweezer setup were in the range of a few nanonewtons and hence on the same order of magnitude as forces generated by cells during migration and adhesion^49^. Therefore, the magnetic tweezer seems to be suitable for investigations of adherent cells.

In fact, we analyzed time- and force-dependent viscoelastic properties of different adherent cell lines at large forces, ranging from 1 to 5 nN. Moreover, we determined the mechanical properties of 3D extracellular matrices, in which these cells possess different capacities to invade^49–52^. Although the displacement of beads coupled to cells and collagen gels varied largely between individual measurements, all displacement curves closely followed a log-normal distribution, as reported elsewhere^29,46^. Beads attached to cells displayed a pronounced non-linear viscoelastic behavior. The non-linear effects appear to be cell type dependent. In general fibroblasts seem to display more non-linear effects compared to breast cancer cells. More precisely, weakly invasive MCF-7 breast cancer cells behave less non-linear compared to highly invasive MDA-MB-231 breast cancer cells. In contrast, weakly invasive PINCH-1^−/−^ cells behave more non-linear compared to highly invasive PINCH-1^fl/fl^ cells.

Although the creep response of all four cell lines still followed a weak power law independently of the amount of the applied force, we observed an increase in the stiffness with increasing force for cancer cells, such as MDA-MB-231 and MCF-7 cells, as well as mouse embryonic fibroblasts, such as PINCH-1 knock-out (PINCH-1^−/−^) cells and controls (PINCH-1^fl/fl^). The observed stress-stiffening effect was more pronounced in the PINCH cell lines than it was in the cancer cell lines. The effect of increasing force on the power law exponent β also showed a pronounced cell type dependent effect. While both mouse embryonic fibroblasts, such as PINCH-1^−/−^ and PINCH-1^fl/fl^ cells, and MCF-7 cells displayed a significant fluidization (i.e. increase of the power law exponent), the power law exponent of MDA-MB-231 cells showed an ambiguous force dependence. Similar results have been reported in earlier studies^30,65,66^. The stiffening and fluidization of cells with increasing force have been mainly linked to passive processes, such as geometrical alignment of stress fibers^30,65^. Since the organization of the cytoskeleton depends on the ambient temperature^67,68^, studies, in which the temperature is controlled only by a heated microscope stage^16,17^ may measure different force dependent effects, such as a force-independent power law exponent^17^, compared to the results presented in this study. Due to the lack of precisely controlled temperature, it is not possible to discern whether these observed differences are due to the different cell lines investigated or are simply an artifact of unstable temperatures. This emphasizes the importance of the incubation chamber employed in this study, which ensures stable conditions during the measurements. In general, stiff and elastic cells, such as the less invasive MCF-7 breast cancer cells, stiffen less and fluidize more compared to soft and fluid-like cells, such as highly invasive MDA-MB-231 breast cancer cells.

Similarly, the repeated application of the same amount of force lead to a cell type dependent effect on both stiffness and fluidity of the investigated cell lines. The stiffness increased for both mouse embryonic fibroblast cell lines, i.e. PINCH-1^−/−^ and PINCH-1^fl/fl^ cells. For both cancerous cell lines, such as MCF-7 and MDA-MB-231, stiffness remained unaffected by the repeated force application. Earlier studies reported a stiffening effect on cells after the application of multiple force pulse sequences^69–73^. Depending on the investigated cell line, a softening may also be observed^69^. However, the duration of the pulses as well as the time between consecutive pulses used in the literature are generally much longer than the 2 s on and 2 s off sequence employed in this study. In fact, stiffening in J774 mouse macrophages has been reported to occur only at pulse lengths longer than 10 s^43^. The stiffening effect has been attributed to active remodeling of focal adhesions the actin skeleton in response to cyclic force application^70,71,73^. Therefore, we propose that the stiffening effect may depend on the time scale of the applied force pulse sequence and the cell’s ability to actively remodel the cytoskeleton and recruit actin in that timescale^71,72^. This is supported by the fact that a significant stiffening effect in bovine aortic endothelial cells only occurs after about 30 s^70^. Therefore, it is plausible that the observed absence of stiffening effects in MCF-7 and MDA-MB-231 cells is due to a slower remodeling compared to mouse embryonic fibroblasts, such as PINCH-1^−/−^ and PINCH-1^fl/fl^ cells. Since active processes, such as the remodeling of the cytoskeleton, may depend strongly on the ambient temperature^67,68^, the employed temperature control in the presented magnetic tweezer setup is crucial to measure the creep curves of cells in response to repeated force application in a reliable manner. Therefore, the reported opposite behavior of cell stiffening with repeated force application may also be related to the lack of a proper temperature control in these studies^17^.

These findings show that living cells, such as breast cancer cells, can actively adapt their stress stiffening and fluidization behavior to the applied external forces. Hence, permanent plastic deformation may occur, if the cells reach their critical point for plastic deformation and bond rupture events occur. However, the permanent plastic deformation may depend on the individual cell type and the specific malignant state of cancer cells. These results are in line with previous AFM measurements of the adhesive breast cancer cells MDA-MB-231 and MCF-7^49,74,75^. Whether the results on the cell mechanics of PINCH-1^−/−^ cells and PINCH-1^fl/fl^ cells can be obtained with other biophysical techniques need to be investigated in future studies. However, since optical cell stretching experiments with ILK^−/−^ cells and ILK^fl/fl^ cells revealed that the less invasive ILK^−/−^ cells are softer (more deformability) in the non-adhesive state^53^, we suggest that the PINCH-1^−/−^ cells will also be softer and more deformable.

The different mechanical properties of the investigated cell lines may play an important role in their invasive behavior. In earlier studies, a direct correlation between stiffness and malignancy of cancer cells has been established and suggested as a reliable biomarker for malignancy^76–78^. Invasive MDA-MB-231 cells are reported to be softer and more fluid than less invasive MCF-7 cells^49,74,75^. These properties may allow them to squeeze through tight constrictions more easily. This mechanical behavior has also been observed in this study. MDA-MB-231 cells are softer and more fluid-like than MCF-7 cells, irrespective of force magnitude or repeated force application. Therefore, the presented magnetic tweezer setup seems to be a promising addition in the toolkit to reliably probe the mechanical properties of cells and further investigate the correlation between invasiveness and cell stiffness.

In addition, we demonstrated that 3D extracellular matrix mechanics, such as matrix stiffness (elastic modulus), can be measured with the environmental chamber equipped magnetic tweezer device. Beads attached to collagen gels, displayed even increased non-linear behavior compared to the four cell types. In addition, we found that denser 3.0 mg/ml collagen matrices were significantly stiffer than looser 1.5 mg/ml collagen matrices, which is comparable to other biophysical techniques, such as Atomic force microscopy (AFM)^60,79,80^, plate rheology^51,62,81^ and MRI^82^. These two concentrations are commonly employed in 3D matrix invasion assays. Although the crosslinking of these matrices increased their stiffness, it did not significantly affect their viscoelastic state, i.e. the power law exponent. However, collagen concentration had a pronounced effect on the power law exponent, as denser matrices displayed a significantly increased fluidity compared to less dense matrices. This finding was in line with several computational models and experiments with crosslinking enzymes, such as transglutaminase^83,84^. The observed fluidization (increase of the power law exponent) of the collagen matrices during the relaxation period may be attributed to rearrangements of the network structure during the stretching, such as the breakage of weak non-covalent crosslinks^85,86^. These structural rearrangements dissipate energy and thus lead to the observed incomplete recovery.

For topological investigations of collagen gels, 3D cubes with an edge length of 100 µm were recorded using a CLSM with an x–y resolution of 2048 × 2048 pixels and about 400 stacks in the z-direction. Therefore, the recorded images had a resolution of 0.13 µm/px in the x–y plane and 0.25 µm in the z-direction. A deconvolution was applied to the images to analyze the pore size on a length scale below the diffraction limit of the system. For computational efficiency, the deconvolved images were rescaled to 37.5% of their original size^60^. Post-deconvolution and multiple post-processing steps of the fluorescent CLSM images of collagen matrices allow analysis with length scales considered to be below the optical diffraction limit. From the investigation of the topological properties of the matrices, we found that fibronectin seems to align the fibers of both 1.5 mg/ml and 3.0 mg/ml collagen matrices. In 3.0 mg/ml collagen gels the crosslinking with fibronectin and thus the fiber alignment leads to a significant increase in pore size. However, crosslinking with fibronectin did not affect the pore size of 1.5 mg/ml matrices significantly, possibly due to the lower density of collagen fibers in these gels. The observed fiber alignment may be linked to the higher stiffness of fibronectin-crosslinked gels, since the crosslinked fibers can respond less freely to the applied stress in the magnetic tweezer measurements.

Similar to cells, the displacement of beads coupled to collagen gels closely followed a log-normal distribution, as reported elsewhere^29,46^. More precisely, due to the small size of the super paramagnetic beads, the mechanical properties of collagen gels were probed on a length scale that seems to be more relevant to cells than probing with other techniques, such as AFM. Hence the magnetic tweezer technique seems to be suited for determining local matrix properties. Additionally, the shear stress applied by the magnetic tweezer was similar to the stresses generated by cells during their migration and invasion through extracellular matrices.

The magnetic tweezer setup presented in this work is a powerful tool to investigate nonlinear viscoelastic properties of a wide variety of cells and soft biophysical materials. By investigating the mechanical differences of various cell types in controlled environmental conditions, it provides an important and reliable contribution in the quantitative phenotypic characterization of cellular processes based on mechanical properties, such as stiffness and fluidity. These parameters are important for the mechanical interaction between cells and the extracellular matrix or tissue environment in normal physiological processes including mechanosensing processes, tissue development and wound healing processes or pathological processes such as cancer, acute and chronic inflammatory responses.

## Key findings (impact on science)

- Improvement of Magnetic Tweezer technique by incorporation of an incubation chamber with temperature and CO_2_ control.
- Cell and matrix mechanics are both accurately described by a weak power law.
- Non-linear viscoelastic properties of cancer cells and fibroblasts are cell line dependent.
- Fibronectin crosslinking affects stiffness of collagen gels but not their viscoelastic state.
- Fibronectin crosslinked fiber alignment evoked a strain stiffening effect of the collagen gels.

## Data Availability

The datasets generated during and/or analyzed during the current study are available from the corresponding author on reasonable request.

## Abbreviations

AFM: Atomic force microscopy
CLSM: Confocal laser scanning microscope
